# Why is tick-borne encephalitis increasing? A review of the key factors causing the increasing incidence of human TBE in Sweden^a^

**DOI:** 10.1186/1756-3305-5-184

**Published:** 2012-08-31

**Authors:** Thomas GT Jaenson, Marika Hjertqvist, Tomas Bergström, Åke Lundkvist

**Affiliations:** 1 Medical Entomology Unit, Department of Systematic Biology, Evolutionary Biology Centre, Uppsala University, Norbyvägen 18d, Uppsala SE-752 36, Sweden; 2 Department for Analysis and Prevention, Swedish Institute for Communicable Disease Control, SMI, Stockholm, Sweden; 3 Department of Infectious Medicine, Sahlgrenska Academy at University of Gothenburg, Gothenburg, Sweden

**Keywords:** *Ixodes ricinus*, *Capreolus capreolus*, *Myodes glareolus*, *Vulpes vulpes*, Bank vole, TBE Epidemiology, Roe deer, Sweden, Tick-borne encephalitis virus

## Abstract

The highest annual incidence of human tick-borne encephalitis (TBE) in Sweden ever recorded by the Swedish Institute for Communicable Disease Control (SMI) occurred last year, 2011. The number of TBE cases recorded during 2012 up to 6th August 2012 indicates that the incidence for 2012 could exceed that of 2011. In this review of the ecology and epidemiology of TBE in Sweden our main aim is to analyse the possible reasons behind the gradually increasing incidence of human TBE during the last 20 years. The main TBE virus (TBEV) vector to humans in Sweden is the nymphal stage of the common tick *Ixodes ricinus*. The main mode of transmission and maintenance of TBEV in the tick population is considered to be when infective nymphs co-feed with uninfected but infectible larvae on rodents. In most locations the roe deer, *Capreolus capreolus* is the main host for the reproducing adult *I. ricinu*s ticks. The high number of roe deer for more than three decades has resulted in a very large tick population. Deer numbers have, however, gradually declined from the early 1990s to the present. This decline in roe deer numbers most likely made the populations of small rodents, which are reservoir-competent for TBEV, gradually more important as hosts for the immature ticks. Consequently, the abundance of TBEV-infected ticks has increased. Two harsh winters in 2009–2011 caused a more abrupt decline in roe deer numbers. This likely forced a substantial proportion of the “host-seeking” ticks to feed on bank voles (*Myodes glareolus*), which at that time suddenly had become very numerous, rather than on roe deer. Thus, the bank vole population peak in 2010 most likely caused many tick larvae to feed on reservoir-competent rodents. This presumably resulted in increased transmission of TBEV among ticks and therefore increased the density of infected ticks the following year. The unusually warm, humid weather and the prolonged vegetation period in 2011 permitted nymphs and adult ticks to quest for hosts nearly all days of that year. These weather conditions stimulated many people to spend time outdoors in areas where they were at risk of being attacked by infective nymphs. This resulted in at least 284 human cases of overt TBE. The tick season of 2012 also started early with an exceptionally warm March. The abundance of TBEV-infective “hungry” ticks was presumably still relatively high. Precipitation during June and July was rich and will lead to a “good mushroom season”. These factors together are likely to result in a TBE incidence of 2012 similar to or higher than that of 2011.

## Review

### Background

**Figure 1:**
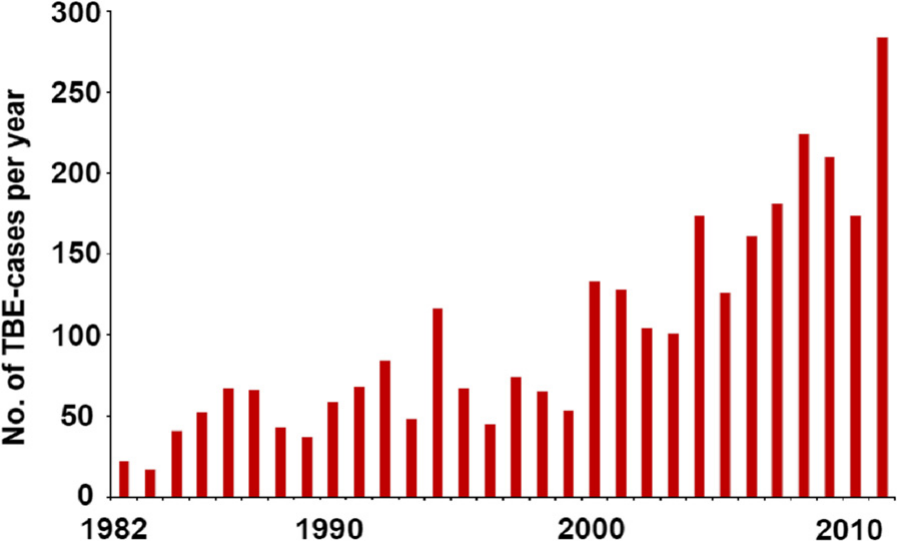
Total numbers of reported human TBE cases in Sweden each year for the 30-year period 1982–2011.

In 2011, 284 people in Sweden developed tick-borne encephalitis (TBE). This is the highest TBE incidence for any single year ever recorded by the Swedish Institute for Communicable Disease Control, Stockholm, Sweden (SMI; Figure 1). Germany, Austria and Finland also recorded exceptionally high numbers of TBE cases in 2011 [1-3]. Based on the numbers of cases recorded for the period from 1st January - 6th August (N = 97 for 2011 and N = 105 for 2012) the annual total TBE incidence for 2012 may exceed that of 2011. In this article we give an overview of the ecology and epidemiology of the TBE virus (TBEV) infection in Sweden. In particular, we analyse how climate change with increasing environmental temperatures and changing tick host abundances have gradually increased the abundance and enlarged the geographic range of the tick *Ixodes ricinus* in Sweden and how these factors have resulted in gradually increasing numbers of human TBE cases since the 1980s.

TBE was first diagnosed in Sweden in 1954. Holmgren and Forsgren [4] reported that TBE was concentrated to the archipelago and coastal areas around Stockholm and Lake Mälaren; about 85% of 1,116 reported human TBE cases from 1956 to 1989 were observed in the county of Stockholm. They also found that the geographical range was remarkably constant over time and that in some areas conspicuous clustering was evident [4].

Ticks are generally considered to be the only arthropod vectors of the TBEV. In Europe the common tick, *I. ricinus,* is the main vector of the TBEV to humans [5-9]. In recent decades this tick species has become very abundant in continental Europe [9], in the UK and on the Scandinavian Peninsula [10,11]. In nearly all regions of northern Europe *I. ricinus* accounts for almost all tick infestations on humans, dogs, cats, horses, cattle and deer [10]. In Sweden *I. ricinus* is considered to be the only vector species for TBEV. All TBE viruses from Sweden have been classified as the European (Western) subtype [11,12], T. Bergström, unpubl. results]. *I. persulcatus,* which is closely related to *I. ricinus,* has so far only been found once in Sweden, specifically a nymph on a warbler captured on the island of Stora Fjäderägg in the Bothnian Sea in May 1992 [10]. About 100 km east of Stora Fjäderägg, in the Archipelago of Kokkola on the west-coast of Finland a permanent population of *I. persulcatus* was discovered in 2005 [13]. Later, this species was detected in a few other localities in Finland, where it is often sympatric with *I. ricinus*, and transmits all three subtypes of TBEV [14]*.* All three subtypes also occur in Latvia, Estonia and in Russia [14-17].

Aside from *I. ricinus* and *I. persulcatus* several other tick species are also competent TBEV vectors [8]. However, natural transmission cycles depend mainly or only on the two *Ixodes* species [8]. The possibility that other arthropods besides ixodid ticks, e.g. fleas and mites, may be enzootic TBEV vectors needs further investigations [18,19]. In the following, we use the terms "tick" and “ticks” as synonymous with *Ixodes ricinus*. In some countries, in addition to tick-borne transmission, humans are occasionally infected with the virus by consuming unpasteurized milk or other dairy products from goats, sheep or cattle [20].

On the Swedish mainland and in much of the rest of Europe it is predominantly roe deer (*Capreolus capreolus*), which are the most important reproductive hosts (= tick maintenance hosts) [21], which implies that the adult ticks feed mainly on such mammals. Deer and other medium- and large-sized mammals are also important meeting and mating sites for the sexually mature ticks, as well as blood hosts for the tick larvae and nymphs [10,22].

**Figure 2:**
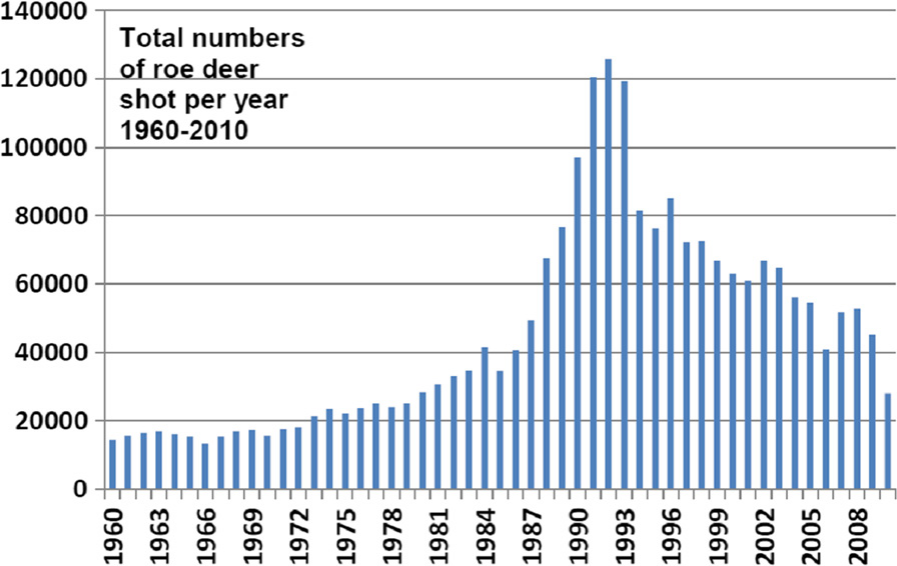
**Total numbers of roe deer shot each year during 1960–2010 in six counties (Stockholms, Uppsala, Södermanlands, Östergötlands, Skåne and Västra Götalands län) with relatively high incidences of human TBE.** Hunting data from Dr Jonas Kindberg, Wildlife Monitoring Unit, Swedish Association for Hunting and Wildlife Management.

In some areas other ungulates [10,20,22,23], and on a few islands, the varying hare (*Lepus timidus*) [24,25], are the main maintenance hosts. The roe deer and *I. ricinus* spend most of their lives in the same vegetation types [26-28]. These are usually deciduous or mixed woodland or forest habitats interspersed with elements of open land. From the 1980s until recently, the roe deer population on the Swedish mainland has been exceptionally high [27,28]; (Figure 2). The large number of roe deer and their exceptional dispersal potential [27] are presumably the main factors that contributed to the increased tick numbers and extended geographic range of the tick population in Sweden during recent decades [23]. The relatively new occurrence of ticks in central and northern Norrland (northern Sweden) is likely a result of the roe deer’s spread there [23,27,29].

The sharp increase in the number of ticks and the extension of the tick's geographic range [23] have increased the risk that tick-borne infections will be transmitted more frequently to humans, even in areas where the infections did not previously exist [23,29]. Roe deer can harbour *Anaplasma phagocytophilum*[30] and *Babesia venatorum*[31] and are presumably often infested with *I. ricinus* that are themselves infected with *Borrelia burgdorferi* s.l*, Anaplasma phagocytophilum, Rickettsia helvetica, Babesia* spp. and the TBE-virus [23]. Therefore, roe deer are likely to play an important role in the dispersal to new locations of ticks infected with such pathogens.

**Figure 3:**
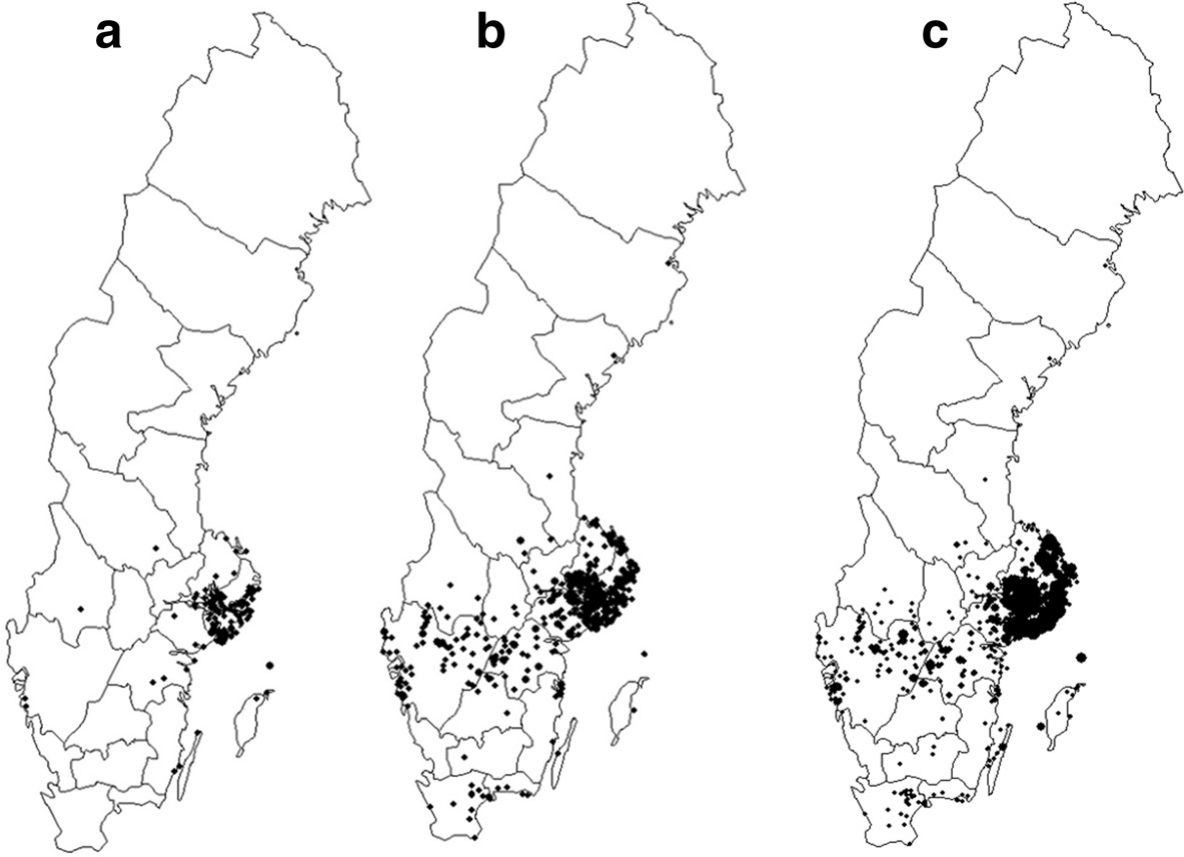
**Each black dot on the maps represents a locality where one or more persons are presumed to have contracted the TBE virus infection.** The left map (**a**) shows the probable places of infection of all domestic TBE cases (n = 236) recorded by SMI during the 5-year period 1987–1991. The central map (**b**) shows the corresponding data 20 years later, i.e. all domestic TBE cases (n = 940) recorded during 2007–2011. Each black dot on the right map (**c**) represents a locality where one or more persons (N = 2550 human TBE cases) are presumed to have contracted the TBE virus infection during the period 1986–2011.

Since its first discovery in Sweden, the annual incidence of TBE has steadily increased. In the 1990s, there were about 60–80 cases/year, except in 1994 when 114 cases were confirmed. Since 2000 there have been >100 cases reported each year (Figure 1). Moreover, the “endemic area” expanded from 1987–1991 to 2007–2011 (Figure 3 a-b) with increased incidences recorded particularly in the south-west-Swedish provinces (landskap) of Västergötland, Dalsland and Bohuslän [32,33]. In this region the first local case was diagnosed in 1997 where after the incidence has gradually risen to a yearly incidence of 2/100.000 inhabitants in 2011. Also in southernmost Sweden, in the provinces of Småland, Blekinge and Skåne (Scania), new localities where people contracted TBE have been recorded [34]. These provinces are all located far from the previously “endemic” area around Stockholm and Lake Mälaren.

In 2007 the first TBE case was recorded from the province of Dalarna. At that time this was the northernmost record for Sweden [35]. In the following years, a few more TBE cases were recorded from Dalarna and even further to the north. Figure 3 a-b shows the presumed places of infection of the human TBE cases recorded by SMI during two 5-year-periods; Figure 3a (left map) shows the places in Sweden where people diagnosed with TBE during 1987–1991 presumably had been infected with the virus. This was the time period during which the Swedish roe deer population had its highest recorded peak, with presumably more than 1 million individuals. Figure 3b (central map) shows the corresponding places where people were TBEV infected during 2007–2011, i.e. 20 years later. The geographic range of the area with localities where people had contracted the virus, was significantly larger and also contained many more TBE cases in 2007–2011 (N = 1,073; mean ± S.D. = 214.6 ± 43.9 cases/year) compared to the period 20 years earlier N = 272; mean 54.4 ± 13.8 cases/year; (P = 0.0007, t = 7.79, d.f. = 4.79, Satterthwaite’s method ). Although the TBE endemic area has expanded and *I. ricinus* now occurs in suitable habitats all over Götaland, in most of Svealand and along the coast of Norrland [23], the majority of the Swedish TBE cases still originate from the Stockholm area, i.e. eastern south-central Sweden (Figure 3b-c). In Götaland, the spread of TBE clearly followed an east-to-west expansion over time. The human cases were initially concentrated to areas near the great lakes Vättern and Vänern, but soon new hot spots emerged at several locations along the coast of Bohuslän and Västergötland, with a later local spread around these established sites. In this region the spread of human TBE consisted of two patterns; one of “leaps” over considerable distances from and between the great lakes in a westward direction towards the Sea of Skagerrak, and the other one as a progressive expansion of already established sites.

The establishment of new TBEV foci far away from the previously “endemic area” may be due to migratory TBEV-infected birds carrying the virus to new locations [36] and to the transportation of TBEV-infected ticks on birds [37,38]. However, adult *I. ricinus* ticks rarely infest birds, with the exception of large ground-dwelling birds such as pheasants (*Phasianus colchicus*). This behaviour reduces the efficiency with which birds may directly or indirectly contribute to the creation of new TBEV foci. Birds have been considered to be incompetent hosts for transmission of the TBEV to ticks [8,37,39]. The finding of TBEV-infected *I. ricinus* larvae on migratory birds [37], however, suggests that these larvae had been infected while feeding on their respective avian hosts - a tree pipit (*Anthus trivialis*) and a European robin (*Erithacus rubecula*). Thus, at least some bird species may be competent hosts for TBE-virus transmission to ticks - either by viraemic transmission or by non-viraemic transmission or by both modes (see the section below on co-blood feeding on non-viraemic hosts). Later in this text we argue that migrating roe deer also could have played and still are playing a significant role in the spread of TBEV and in the founding of new foci of TBEV as well as other tick-borne pathogens.

### *Ixodes ricinus* nymphs are the main vectors of TBEV to humans

The tick’s life cycle consists of three active stages: larva, nymph and adult. In each active stage the tick generally ingests blood only once; then moults to begin the next life stage. Each stage lasts for 1–2 years, sometimes up to 3 years [40,41]. Based on studies from England and Ireland [40] and Germany [41] we can estimate that the temperature-dependent development cycle from egg to egg-laying female in southern Sweden takes at least 4 years and in coastal Norrland about 6–7 years. The nymphs of *I. ricinus* are the main vectors of TBEV to humans [6,7,39,40,42,43]. In *I. persulcatus,* it is the adult females – not the nymphs – which usually transmit the infection to humans. Adult females of both species and transovarially infected larvae can also transmit the virus to host animals [8,43]. Adult male ticks rarely ingest host blood and are therefore not important for the direct transmission of TBEV to humans. However, indirectly male ticks may be of importance in the epidemiology of TBEV, since the virus may be transferred from male ticks to female ticks during copulation via infective male saliva and/or seminal fluid [44]. The virus remains virulent in the infected tick for at least several months and presumably for more than a year. Generally, the virus is transferred transstadially, i.e., from one tick stage to the next stage, e.g., from nymph to adult.

### TBEV transmission to ticks from viraemic small mammals

To comprehend the TBEV transmission cycle, it is fundamental to know how the virus usually infects susceptible ticks. This fact can then be used to understand how the tick's hosts, in combination with temperature and humidity close above and in the uppermost soil layer, can affect the number of TBEV-infected ticks.

Transovarial TBEV transmission, which is the transfer of the virions via the eggs from an infected adult female tick to her offspring, sometimes occurs but usually at such a low frequency [8,43] that it cannot explain how the TBEV infection can persist in a particular focus year after year [43]. Previously it was thought that the main mode of transmission of TBEV to ticks was that they became infected by “systemic infection” while blood-feeding on viraemic rodents [5]. However, the virus is generally only present for a few days at a sufficiently high concentration in the blood of infected rodents to enable the infection of ticks. This may not be enough time or high enough dose to infect a sufficient number of ticks to maintain the transmission cycle of TBEV [8,45-47]. However, recent field and laboratory research in Germany shows that TBEV in the common vole *Microtus arvalis* is detectable in different organs for at least 3 months, and in the blood for 1 month after infection [47]. In the same study, ten per cent of all rodents investigated were positive for TBEV and the bank vole *Myodes glareolus* showed a high infection rate in all localities investigated [47]. If it were proven that the virus concentration in the blood of these viraemic small mammals is sufficiently high to infect ticks, then TBEV transmission from viraemic small rodents to ticks could be of great importance in maintaining the virus in nature.

Insectivores and rodents may also act as TBEV reservoirs since they may maintain the virus latently during the winter. In such TBEV-infected small mammals a viraemia may develop [5]. As noted previously, whether such a viraemia has a sufficient virus concentration to be infective for feeding ticks during the following season needs further investigations.

Large mammals such as deer, goats, sheep and cattle are, in comparison with small mammals, considered to be viraemic for a very short period of time [8,20,48]. In conclusion, the viraemic mode of transmission by ungulates is probably of little importance in the maintenance of TBEV. Viraemic transmission of TBEV from small mammals to feeding ticks may, however, be of greater significance for the maintenance and spread of the virus in nature than has been generally believed.

### Infection of ticks co-blood feeding on non-viraemic hosts

A very important mode of TBEV transfer among ticks is termed non-viraemic transmission and takes place when ticks are co-feeding on small rodents, mainly of the genera *Myodes* and *Apodemus*[46,49-55]. Here, virions are transmitted to one or more susceptible blood-feeding tick(s) - via the tick host’s phagocytic migratory white blood cells - from one or more infective ticks that feed close to the susceptible ticks on the same host animal [55]. Transmission of virions in this manner usually occurs from nymphs to larvae [46,55]. Typically, the number of larvae is much greater than that of nymphs [56]. The simultaneously blood-sucking larvae and nymphs most often feed in groups close together on the ears or other parts of the head of a rodent such as a yellow-necked field mouse (*Apodemus flavicollis*), wood mouse (*A. sylvaticus*) or bank vole (*Myodes glareolus*). Many of the virus particles are quickly eliminated by the host’s phagocytic leucocytes [53-55] without any strong viraemia being developed in the host. Thus, if one or more tick larvae are attached in the host’s skin adjacent to the infecting nymph(s) then some of the virus-infected leucocytes may be sucked up by susceptible larvae.

Roe deer, sheep, goats, cattle and other important mammalian tick hosts and birds have not (yet) been proven to be competent hosts supporting non-viraemic transmission between co-feeding ticks [8,46]. Roe deer, goats, sheep and cattle develop a strong antibody response to the TBEV infection [48,57-59]. The presence of antibodies against TBEV, however, does not preclude that these large mammals may be competent hosts for non-viraemic TBEV transmission among co-feeding ticks as well. This is true particularly in view of the oftentimes very high tick infestation rates, especially on the ears and other parts of the head of deer. It is more likely though that small - and medium-sized, densely infested tick hosts – such as rodents and hares - are more important in the non-viraemic pathogen transmission than larger-sized hosts. Due to the limited space available on small rodents any tick larvae would be “forced” to feed very close to potentially infective ticks presumably rendering any virus transmission among co-feeding ticks relatively efficient. Investigating the potential role of roe deer, other cervids and domestic ungulates in the possible maintenance and spread of the TBEV may be rewarding.

### Simultaneously host-seeking larvae and nymphs

In order for nymphs and larvae to be able to attach to the same host animal, for example a small rodent or shrew, a prerequisite is that both tick stages are active in the same time period. A cold winter followed by a relatively rapid rise in spring temperatures is considered to optimize the simultaneous activation of larvae and nymphs [39,56,60]. Such a weather situation will enable them to parasitize the same host individual at the same time in spring. In contrast, if spring temperature increases relatively slowly, then the nymphal activity peak will occur several weeks earlier than the larval activity peak. This is because the nymphs become activated to host seeking and will infest hosts at a lower temperature threshold, around 5–7°C, than the considerably smaller larvae, which begin questing at about 10°C. In the case of slow warming in spring, nymphs, in comparison to larvae, will infest hosts earlier. However, in southern Sweden, nymphs have a seasonal pattern of questing and infesting small rodents similar to those of larvae, although the nymphal activity usually starts somewhat earlier in spring [61]. Field studies carried out in woodland biotopes in central and southern Sweden from 1990 to 2011 confirm that many tick larvae are active simultaneously with nymphs from June to September [TGT Jaenson, unpublished data]. Therefore, non-viraemic transmission between co-feeding subadult ticks on rodents and possibly on other terrestrial vertebrates may occur in Sweden even in the summer and early autumn.

Another factor that may influence the proportion of nymphs that will infest small mammals is the water content of the air layer close to and in the ground [62,63]. Ticks are highly sensitive to dehydration and therefore prefer to reside in highly humid environments. This applies especially to the tiny larvae but also to the nymphs and to a lesser degree to the somewhat larger adult ticks. The larvae stay almost exclusively on or just below ground level. The nymphs also spend proportionally more time questing on or closer to the ground. When the saturation deficit of the air increases in dry weather, the nymphs spend proportionally more time questing on or closer to the ground. This behaviour increases the likelihood that the nymphs, just like the larvae will encounter and attach to small rodents or shrews. Due to their proximity to the ground, these mammals are thus at the same level as ticks during dry weather. In a humid microclimate, nymphs often climb higher up in the vegetation. Then the likelihood that they will attach to a small mammal is reduced while their potential to attach to a larger mammal, such as a deer, increases.

### The roe deer’s spread northwards promoted the expansion to the north of the vector and the virus

To understand all fundamental factors that have promoted the tick’s increasing abundance and range expansion as well as the increasing incidence of TBE both northwards and westwards in Sweden [23], it is important to know that the adult ticks most frequently feed on relatively large mammals, e.g. deer, hares, dogs and cats, and large birds such as pheasants. The roe deer and other cervids are important blood sources also for the larvae and nymphs, but are especially important for the adult female ticks [10,22,23,40,42,64]. Cervids and some other large mammals are also a meeting and mating place for the reproducing adult ticks [10]. Evidence for these claims includes the substantial numbers of larvae, nymphs and adult ticks of both sexes commonly found on deer in Sweden during the spring, summer and autumn [10,22,23,64]. Adult *I. ricinus* are almost never found on small birds [10,37,38,65] or on small mammals [10,22,42,61] (Table 1).

**Table 1:** **Infestation of mammals by larvae, nymphs and adult females of *Ixodes ricinus* **

	**Larvae**	**Nymphs**	**Females**	**L:N:F ratio**	**Range (all tick stages)**	**Number of hosts examined**
Myodes glareolus*	34	0.9	0	38:1:0	1-219	106
*Apodemus* spp.*	60	1.5	0	40:1:0	4–451	50
Capreolus capreolus*	265	93	30	2.8:1:0.3	428–2072	37
*Lepus timidus* *	630	255	13	2.5:1.0:0.05	458–1725	8
Lepus timidus**	412	53	6	7.8:1:0.1	87-2374	15

Larvae, Mean no. larvae; Nymphs, Mean no. nymphs; Females, Mean no. adult *I. ricinus* females; L:N:F, Larvae : Nymphs : Females; *, from provinces of Uppland and Södermanland on the Swedish mainland; **, from Gotska Sandön, which is an isolated island in the Baltic Sea.

Data from Tälleklint & Jaenson [22].

In Sweden there is considerably more wildlife today than 50 years ago [66]. From the 1980s until the winter of 2009–2010 roe deer were common in almost all of Sweden [27-29,66] except on some islands where the mountain hare [24,25] was and still is the main host for high-density tick populations. The number of roe deer at present, i.e. August 2012, is rapidly increasing [67]. Additionally, the moose (*Alces alces*) population in Sweden has been increasing for several decades [66]. In some places there are even expanding populations of red deer (*Cervus elaphus*) and fallow deer (*Dama dama*) [66], which are locally important blood hosts for *I. ricinus* although not yet of the same magnitude as the roe deer. Other large mammals which are also potential hosts of adult *I. ricinus,* such as wild boar (*Sus scrofa*), mouflon (*Ovis musimon*) and badger (*Meles meles*, have increased their population sizes in Sweden during the last 50 years as well [66].

Because the roe deer has been so abundant in the same habitats that are optimal for ticks, we propose that the roe deer has played a particularly important role in the tick's expansion in Sweden. There is probably no other vertebrate species that has been so important for the tick’s propagation and range extension on the Swedish mainland as the roe deer during the past half century. Its exceptional ability to spread, especially along the north Swedish river valleys [27], is a factor not previously mentioned as being instrumental for the tick's rapid expansion in the north.

### Increasing tick abundance and TBEV prevalence promoted by higher environmental temperatures

Climate change is a fundamental factor that directly and indirectly has contributed to the increasing abundance of the roe deer and thus also to the tick’s increasing abundance, especially in the north. The trend towards warmer winters and longer growing seasons with earlier springs and later autumns has undoubtedly been beneficial for the survival and proliferation of the roe deer and the tick [23,29].

Based on detailed records on the incidence of TBE in the Stockholm County for the period from1960 to 1998 Lindgren and Gustafson [68] analysed if there was a link between climate change and TBE incidence which increased substantially in the 1980s. They found that increases in TBE incidence were significantly related to a combination of two consecutive mild winters, relatively high spring temperatures and a prolonged mild autumn in the year prior to the incidence year, and temperatures allowing tick activity early in the incidence year. These results are consistent with those of Jaenson and co-workers [69] and Jaenson and Lindgren [29] who showed that the tick is usually abundant in areas where the vegetation period is at least 180 days per year. The vegetation period is the number of days between the end of the first continuous 4-day period with a 24-h mean temperature >5°C and the beginning of the last 4-day period with a 24-h mean temperature >5°C. The tick may be present but is not abundant when the vegetation period is 160–180 days per year, and the tick is nearly always absent from areas with <160 vegetation days/period [69]. Daily mean temperatures >5°C correspond to the threshold temperature for host-seeking activity of nymphal *I. ricinus* in Sweden (TGT Jaenson, unpubl. data, [69]). Consequently, the increasing temperatures with a gradually longer vegetation period are likely to favour tick activity, tick abundance and the tick’s geographic range. In areas of southwest Sweden (Halland, Västergötland, Bohuslän, Dalsland, Värmland), where summer mean temperatures are lower than in southeastern Sweden (Öland, Gotland, Småland, Östergötland, Södermanland, Uppland) *I. ricinus* was usually relatively rare before the 1990s. In South Sweden, particularly in the southwest ticks appear to have increased in abundance during the 1990s and become much more common [23]. In the areas of Sweden where ticks occurred in low-density populations prior to the 1990s TBE was usually considered “not endemic” and was only recorded there very occasionally or not at all. During the late 1990s the incidence of TBE, however, began to increase rapidly in such previously “non-endemic” areas [32-34]. It is likely that the TBEV infection is “favoured” by an abundant vector population, which facilitates increased transmission of the virus among densely tick-infested small mammals.

The environmental temperature also has a direct effect on the invertebrate vector and on the virus within the vector. The extrinsic incubation period (EIP) is an important concept in the epidemiology of many mosquito-borne and other insect-borne arboviruses and microparasites. However, as emphasised by Nuttall and Labuda [8], since the EIP is unlikely to exceed the comparatively long moulting period in ixodid ticks it may not be important for virus transmission by such vectors. Yet, the temperature influences TBEV development and propagation in *I. ricinus*: Recently Magnus Johansson’s group at Södertörn University, Sweden [70] detected and described a temperature dependent structural RNA rearrangement between open and closed conformations that acts as a temperature-sensitive riboswitch for on/off setting of TBEV translation in the questing tick in nature. In TBEV-infected ticks in nature the viral genome usually exists in a closed form which may – at least partly - explain the low or undetectable virus levels in host-seeking ticks collected in TBE “risk areas” [70]. For a virus strain isolated from Torö near Stockholm the lower temperature breakpoint, 25.4-28.1°C is the most pronounced when RNA was suggested to unfold [70]. Although this process remains to be proven for TBEV during natural infection it is here suggested that a continuous period of several warm days or a brief period of high temperatures will induce RNA unfolding and translation and subsequent viral propagation in the tick. From an evolutionary point of view it should be advantageous for the virus to be present as infective virions in a high a concentration in the tick’s salivary glands, ready to infect a potential host that happens to come close to the questing, virus-loaded tick. The emergence of “new high-risk areas for TBE” in southwest Sweden may thus, at least partly, depend on climate change with more frequent and longer periods of high temperatures during the tick season. Such warm periods are likely to favour development of the TBEV to infective virions in the vector.

### More than one million roe deer in the early 1990s

Cervids are favourite game animals for many hunters who invest money, time and energy in an attempt to establish high-density populations. This is accomplished by several practices, such as winter-feeding the game animals and hunting foxes, lynx and wolves, as well as other measures. This "care" primarily aims to provide rich populations of game animals, specifically various species of deer. Without winter feeding in the north of Sweden the roe deer, for example, could probably not have survived there more than temporarily [27]. Even in southern and central Sweden, many roe deer died during the two cold and snowy winters 2009–2011. Forest grazing of cattle almost ceased during the latter part of the1800s. Because cattle formerly competed with deer for space in woodland and forested areas the abandonment of cattle forest pasture grazing favoured the deer populations as well as tick populations. Also, the many clear-cuts of recent forestry as well as the regulation of deer hunting with hunting-free periods have increased the roe deer population size [27].

The red fox (*Vulpes vulpes*) is a major predator on young deer in southern and central Sweden. However, during the period from 1972–1975 sarcoptic mange due to the scabies mite (*Sarcoptes scabiei*) infested the red fox population of northern Sweden and the infection spread rapidly southwards. Within 10 years more than 50% of the red foxes on the Swedish mainland were obliterated [71]. As a direct consequence, the number of roe deer increased dramatically in the late 1980s. Moreover, in the early 1990s, the winters were mild with little snow, further favouring the roe deer’s winter survival as well as its expansion to the north of Sweden. During 1992–94 there were probably more than 1 million roe deer in Sweden.

When the fox mange began to disappear during the late 1980s the number of foxes increased and in the 1990s fox predation on young deer became more intense. During the 1980s the lynx population had decreased to about a few hundred individuals. Due to its protection by law in 1991–1995, their numbers increased, especially in Norrland and Svealand. The lynx is now another significant factor in the regulation of the Swedish roe deer population.

Due mainly to predation by fox and lynx the number of roe deer gradually declined after the population peak in 1990–94. Yet the roe deer population was so numerous that each year until 2009 hunters managed to shoot more than 100 000 individual deer and in some years more than 200 000 deer [27,66]; Dr Jonas Kindberg, Wildlife Monitoring Unit, The Swedish Association for Hunting and Wildlife Management, personal communication]. Since the roe deer was so numerous in the tick’s primary habitats for at least the last three decades, it was a readily available mating site for the sexually mature ticks as well as a food source, especially for the adult tick females. The result was an increased tick population level — although in the first years the increase was not so obvious and relatively slow due to the lengthy life cycle of *I. ricinus*[40,41,72]. As a consequence, tick numbers became very high in optimal tick biotopes in southern and central Sweden after about a decade. *Ixodes ricinus* is now considered to have reached “pest status” in large areas of Sweden.

The important “public health role” of the red fox are two-fold. First, it is an important predator of young roe deer. By killing deer the fox indirectly reduces the number of ticks produced. Second, the fox is a key small-mammal predator. Many small mammal species are important reservoirs for several tick-borne pathogens, e.g., TBEV, *Borrelia afzelii, B. burgdorferi* and *Rickettsia helvetica.* Thus, the red fox should have a significant role in reducing the density of ticks infected with human pathogens. Support for this view comes from studies in the US where increases in Lyme disease have continued over the past two to three decades, long after the recolonisation of deer, and coincide with a range-wide decline of the red fox, likely due to expansion of coyote populations [73]. The situation in Sweden differs markedly from that in the US. The Swedish fox population, although subjected to intense hunting pressure, has regained its former population level after the severe scabies epizootic [Dr Jonas Kindberg, Swedish Hunting Statistics, Wildlife Monitoring Unit, Swedish Association for Hunting and Wildlife Management].

### Cervids incompetent hosts for *Borrelia burgdorferi* s.l. and the TBE virus

There are significant relationships between roe deer density and abundance of *I. ricinus*[74-79], nymphal abundance and density of *Borrelia-* infected nymphs [69,77,79] and between density of *Borrelia-* infected nymphs and Lyme borreliosis (LB) incidence in humans [80]. However, since roe deer are incompetent hosts (= non-reservoir hosts [21]) for *B. burgdorferi* s.l. [64,81] and presumably also for the TBEV, deer will divert larval ticks from feeding on reservoir-competent hosts to feeding on deer. This will result in a negative relationship between the density of *I. ricinus* nymphs and the density of nymphs infected with *B. burgdorferi* s.l*.*[30,73,77,79]. The relationship between roe deer density and TBEV infection in ticks and rodents has recently been investigated in much detail and presented by Annapaola Rizzoli and her scientific team [74-76,82]. In agreement with the relationship between LB incidence and deer abundance, they found that deer abundance initially has a positive effect on the number of ticks feeding on rodents; then deer abundance reaches a threshold value above which the effect becomes negative since deer appear to divert ticks from feeding on rodents [76,82]. However, Cagnacci and co-workers [76] also showed that the prevalence of TBEV in ticks and rodents had a monotonically negative relationship with deer abundance. The negative relationship between deer density and TBEV prevalence is most likely due to deer being highly “attractive” to the immature *I. ricinus* ticks but at the same time reservoir-incompetent for TBEV thereby diverting questing ticks from TBEV-competent rodents, i.e. what may be termed the dilution effect [76,82]. In other words, at high deer abundance a great proportion of the immature tick population feed on these reservoir-incompetent mammals. This reduces the TBEV transmission intensity from infective nymphs co-feeding with susceptible larvae on small mammals. In such an ecosystem with plenty of deer the transmission of TBEV from viraemic small mammals to immature ticks is also reduced. Reduction of the deer population from a high level of abundance that has resulted in the “production of an abundant tick population” will induce increased feeding on small mammals by the immature ticks. This will consequently lead to an increased prevalence of TBEV infection in both ticks and small mammals. In such an ecosystem the risk for humans contracting the TBEV infection is increased compared to the first example where the abundance of deer is high.

In Sweden the roe deer population gradually decreased from a very high level (>1 million in the early 1990s) to the present, significantly lower, level (about 200,000 deer). At the previous high level the dilution effect by roe deer is considered to have been remarkable so that, not only the adult tick females, but for this analysis even more importantly, a significant proportion of the immature ticks were feeding on the then easily available deer. Only a relatively small proportion of the tick population was feeding on reservoir-competent small mammals. Thus, the proportion of TBEV-infected ticks in the South Swedish *I. ricinus* population should have been relatively small in the early 1990s. With a gradually decreasing roe deer population but still a very abundant tick population each year after the roe deer population peak gradually larger proportions of the larval segment of the tick population were feeding on reservoir-competent small mammals. The result was an increasing proportion of TBEV-infected ticks over the period from the early 1990s to 2012. The abundant tick population with a gradually increasing prevalence of TBEV-infected ticks is reflected in the gradually increasing numbers of TBE cases in the human population (Figure 1).

### Many small rodents in 2010 led to many infective nymphs in 2011

Roe deer numbers have gradually declined since the early 1990s. Therefore, to support a large tick population with blood, small mammals have presumably become increasingly more important as a food source for the immature ticks. Roe deer numbers decreased even more abruptly during and briefly after the two cold and snowy winters of 2009–2010 and 2010–2011. Apart from the harsh weather with deep snow causing malnutrition the decline of the roe deer population was presumably also due to predation by lynx and fox and also by wolves in some areas. In the years 2010–2011, there were few or no roe deer left in some areas, especially in the roe deer’s northern Swedish range. Thus, the most important food source for the tick was no longer as readily available.

The rodent populations, particularly the bank vole population, vary greatly in numbers from year to year, especially in areas of Sweden with deep snow cover. 2010 was a peak year, especially for the bank vole [83]. Since roe deer had decreased in numbers, first gradually and then suddenly, while rodent numbers had increased in 2010 concomitantly with the sudden decrease of roe deer, we presume that tick feeding changed abruptly in 2010 to reflect this shift in host density. Thus, a large proportion of the larval and nymphal segments of the *I. ricinus* population succeeded in attaching to rodents. This likely increased the rate of non-viraemic TBEV transmission from infective nymphs to susceptible tick larvae and of viraemic transmission [47] from TBEV-infective rodents to immature ticks. The result of this was that many tick larvae became TBEV-infected in 2010. Most likely, it was mainly these ticks that in 2011 and 2012 had become nymphs, which managed to suck blood from people, some of whom became infected with the TBEV.

### People's knowledge and behaviour affect their exposure to ticks

The degree of contact between TBEV-susceptible people and TBEV-infective ticks, mainly nymphs, is a main determinant of the proportion of the human population that will become infected with TBEV. People's susceptibility to the infection is of course influenced by immunological factors including whether or not they have been optimally vaccinated. However, people’s knowledge about measures against ticks and tick-transmitted infections may also explain if people will become infected. Weather and climate will influence the occurrence and abundance of edible berries and therefore to a certain extent will dictate how many people will be available to questing ticks. Likewise, rainfall during the summer affects the presence of mushrooms and thus the number of mushroom pickers from August to October.

The amount of TBE virions infecting a susceptible person presumably influences whether the infection will remain as an asymptomatic infection or progress to an overt disease [51]. An important lesson would therefore be that an infective tick, which has the opportunity to remain attached in the person’s skin for a long time, is likely to transfer more TBE virions and possibly other potential pathogens than a tick that is rapidly discovered and removed. If the tick has already had time to form a cement plug around its mouthparts in the person’s skin another important prophylactic measure to avoid a massive infection dose would be to remove the plug as soon as possible. The reason is that such a plug formed by a TBEV-infected tick is likely to contain a relatively high number of virus particles [51].

A TBEV-infective adult tick female is most probably more dangerous than a TBEV-infective nymph. The reason is that the larger female potentially carries more TBE virions (and other pathogens). Fortunately, people usually detect the hungry female adult tick, with her colourful red and black body, more readily than the much less conspicuous hungry nymph. Apart from the female tick’s conspicuous red body, her length is about 3.5 mm whereas the nymph is greyish and smaller, with a body length of about 1.5 mm. However, *I. persulcatus* females attach to people quite often. Therefore, the behaviour of this tick species is more dangerous compared to *I. ricinus*.

## Conclusions

### An “epidemiological chain of triggering factors”

Due to the large deer population during the 1980s and 1990s the Swedish tick population gradually increased. At the turn of the century the tick population in Sweden was presumably larger than ever before. After its peak in the late 1980s and early 1990s the roe deer population level declined gradually until it was suddenly reduced due to the two harsh winters of 2009–2011. During the gradual decline of the roe deer population a gradually larger proportion of the tick larvae and nymphs probably fed on small mammals, which are reservoir-competent hosts for TBEV. Consequently, from the mid-1990s to 2011 a larger proportion of the tick population became infected with TBEV. A reflection of these events is the gradually greater annual incidences of TBE in the human population during the same time period. Climate change and weather events with high temperatures further influenced the infection prevalence in the tick population and therefore also the annual TBE incidence in humans. As we have seen, there were many interacting factors that caused so many people in Sweden to become ill with TBE in 2011 and 2012. Perhaps the main cause of this "epidemiological chain of triggers" was the very high abundance of the tick *I. ricinus* — a consequence of a large roe deer population from the 1980s and early 1990s. The severe winter in 2009–2010 was followed by periods in late spring and summer of relatively high temperatures [84]. Many larvae and nymphs may therefore have co-fed on small rodents, some of which may even have been viraemic. In July and the first half of August 2010 the weather was favourable for larvae and nymphs to co-feed on small rodents.

The population peak of the bank vole in 2010 coincided with a significant decline in the number of roe deer with the result that an unusually large number of tick larvae presumably co-fed with potentially infective nymphs on rodents. This led to an exceptionally large number of larvae having become infected with TBEV that year. Many of these larvae developed to infective nymphs. In the hot and humid year 2011, starting at the end of March 2011 and lasting until the end of December, these nymphs could blood-feed and infect many people with TBEV.

**Figure 4:**
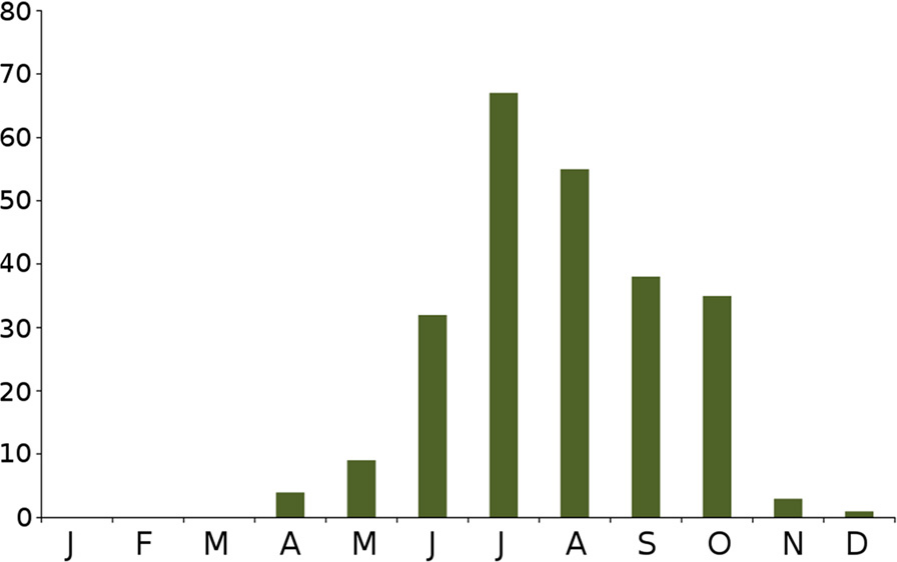
**Total number of reported TBE cases in Sweden during 2011 (N = 284 cases).** The cases are grouped by month of appearance of the first symptoms compatible with TBE.

The reason why the tick attacks on humans and the transmission of TBEV to humans was so frequent in 2011 and 2012 was a consequence of (i) the gradual decline of roe deer numbers from a very high level, (ii) unusually high number of host-seeking ticks being present and (iii) a gradually increasing host-shift by immature ticks from roe deer to small rodents resulting in increasingly greater prevalence of TBEV infection in the tick population. It was also due to the fact that (iv) roe deer and also rodents had decreased in numbers in 2011 and 2012, so that these potential hosts no longer "competed" with humans as potential tick hosts to the same extent as in previous years. Moreover, (v) in 2011 people were more available as potential hosts for questing ticks since people spent more time outdoors in the warm and sunny weather during the unusually early and warm spring and warm and wet summer, and in the mushroom woodlands during the latter part of the year. In 2011 (vi) the vegetation period was exceptionally long and the weather was often warm and humid [85], which permitted nymphs and adult ticks in southern Sweden to begin questing already in late March and thereafter for most days until the end of December 2011 (Figure 4). This made contact between humans and the many nymphs, some of which were TBEV-infective, unusually frequent. The overall consequence was that many people were tick-bitten in 2011, a minor proportion of whom became infected with TBEV and of which 284 persons were recorded as having fallen ill with TBE.

### The present TBE situation in Sweden July 2012

The epidemiological scenario of TBE in Sweden during the last few years would lead to the projection that many cases of human TBE in Sweden will also occur this year. The tick population should have decreased further and be smaller compared to 2011 due to the fact that the deer population has not yet "recovered" substantially after the recent harsh winters. Recent reports, however, suggest that after a mild winter and heavy summer rains, which has resulted in lush vegetation, the deer population is rapidly increasing. However, the infection prevalence in nymphs and adult ticks should still be about as high as in 2011 because roe deer are still not “diverting” ticks from feeding on rodents to the same extent as they did prior to the winters of 2009–2011. In other words, small rodents, being competent reservoir hosts permitting non-viraemic and viraemic TBEV transmission, should be significantly more important for TBEV transmission in 2012 than before 2009.

The mean monthly temperature of March 2012 was among the three warmest March temperatures ever recorded by the Swedish Meteorological and Hydrological Institute (SMHI) [86]. This is likely to have permitted immature ticks to feed on and become TBEV infected from small rodents. Due to high rainfall in the summer of 2012 [87,88] it is likely that, during the “mushroom season” this year, there will be significant close contact between virus-infected vectors and mushroom-picking humans potentially leading to increased virus transmission from infective nymphs to humans. Data up until 6th August 2012 indicates that the TBE incidence in humans will be as high as or higher than that of 2011.

## Endnote

^a^ This is an extended English version of an article published in Swedish only as: Jaenson TGT, Hjertqvist M, Lundkvist Å. [År 2011 toppar TBE-incidensen. Rådjursstammens variation i storlek och vädret är nyckelfaktorer]. *Läkartidningen (Journal of the Swedish Medical Association)* 14th February 2012; 109(7):343–346.

## Competing interests

The authors declare that they have no competing interests.

## Authors’ contributions

TJ collected, reviewed, analysed and synthesised published and unpublished information on the subject and wrote the initial and final versions of the manuscript. MH compiled the data for Figures 1, 3 and 4. TB contributed with unpublished virological and epidemiological data; all co-authors co-revised the manuscript and co-refined the intellectual content of the manuscript. All authors read and approved the final version of the manuscript.
